# Supplementary material for: The causal relationship between the human gut microbiota and pyogenic arthritis: a Mendelian randomization study
Source: Front Cell Infect Microbiol. 2024 Nov 26;14:1452480. doi: 10.3389/fcimb.2024.1452480 (PMC11629706; doi:10.3389/fcimb.2024.1452480)
Supplement: Supplementary file 1 [file DataSheet1.zip › mendelian test/ebi-a-GCST90027646.csv_forest.pdf]

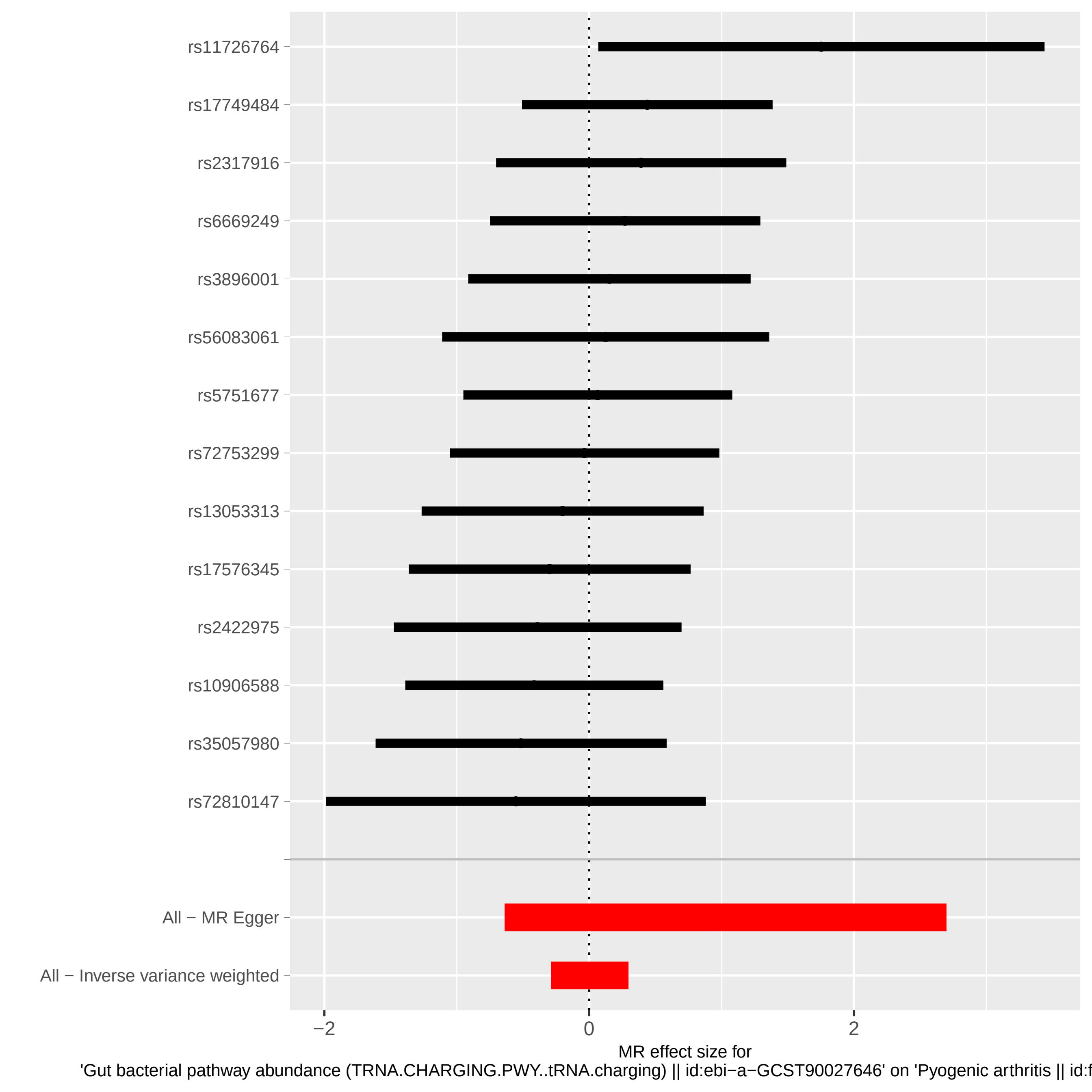
rs11726764
rs17749484
rs2317916
rs6669249
rs3896001
rs56083061
rs5751677
rs72753299
rs13053313
rs17576345
rs2422975
rs10906588
rs35057980
rs72810147
All − MR Egger
All − Inverse variance weighted
−2
0
2
MR effect size for
'Gut bacterial pathway abundance (TRNA.CHARGING.PWY..tRNA.charging) || id:ebi−a−GCST90027646' on 'Pyogenic arthritis || id:f
